# Supplementary material for: Allosteric regulation and crystallographic fragment screening of SARS-CoV-2 NSP15 endoribonuclease
Source: Nucleic Acids Res. 2023 Apr 28;51(10):5255–70. doi: 10.1093/nar/gkad314 (PMC10250223; doi:10.1093/nar/gkad314)
Supplement: gkad314_Supplemental_Files [file gkad314_supplemental_files.zip › Supplementary datasets.zip!/217/Refine_9/01-BUSTER/Cycle-3/shell.01/R_Rfree.html]

# Rxpct factors in resolution bins at cycle 0


|  |  |  |  |  |  |  |  |  |
| --- | --- | --- | --- | --- | --- | --- | --- | --- |
|  | **<d> (Å)**  Rxpct\_Work | Rxpct\_Free | Rxpct\_All | |  |  |  |  |
|  | 16.1098 | 0.3904 | 0.4385 | 0.3921 |  |
|  | 9.3010 | 0.2333 | 0.2786 | 0.2359 |  |
|  | 7.2045 | 0.2018 | 0.2652 | 0.2053 |  |
|  | 6.0889 | 0.2050 | 0.2768 | 0.2083 |  |
|  | 5.3699 | 0.1619 | 0.2426 | 0.1652 |  |
|  | 4.8573 | 0.1499 | 0.1940 | 0.1525 |  |
|  | 4.4681 | 0.1513 | 0.2144 | 0.1545 |  |
|  | 4.1595 | 0.1497 | 0.2184 | 0.1537 |  |
|  | 3.9072 | 0.1605 | 0.2398 | 0.1644 |  |
|  | 3.6958 | 0.1628 | 0.2304 | 0.1658 |  |
|  | 3.5155 | 0.1707 | 0.2016 | 0.1722 |  |
|  | 3.3591 | 0.1761 | 0.2310 | 0.1793 |  |
|  | 3.2220 | 0.1866 | 0.2218 | 0.1886 |  |
|  | 3.1003 | 0.1932 | 0.2148 | 0.1943 |  |
|  | 2.9915 | 0.1923 | 0.2416 | 0.1942 |  |
|  | 2.8934 | 0.2059 | 0.2487 | 0.2082 |  |
|  | 2.8044 | 0.2086 | 0.2356 | 0.2097 |  |
|  | 2.7231 | 0.2094 | 0.2736 | 0.2127 |  |
|  | 2.6484 | 0.2366 | 0.3045 | 0.2410 |  |
|  | 2.5796 | 0.2560 | 0.2785 | 0.2572 |  |
|  | **Overall** | 0.1883 | 0.2404 | 0.1909 |  |

# Rxpct factors in resolution bins at cycle 1


|  |  |  |  |  |  |  |  |  |
| --- | --- | --- | --- | --- | --- | --- | --- | --- |
|  | **<d> (Å)**  Rxpct\_Work | Rxpct\_Free | Rxpct\_All | |  |  |  |  |
|  | 16.1098 | 0.3923 | 0.4397 | 0.3939 |  |
|  | 9.3010 | 0.2337 | 0.2787 | 0.2364 |  |
|  | 7.2045 | 0.2027 | 0.2664 | 0.2062 |  |
|  | 6.0889 | 0.2044 | 0.2763 | 0.2076 |  |
|  | 5.3699 | 0.1616 | 0.2423 | 0.1649 |  |
|  | 4.8573 | 0.1492 | 0.1933 | 0.1517 |  |
|  | 4.4681 | 0.1510 | 0.2150 | 0.1543 |  |
|  | 4.1595 | 0.1493 | 0.2181 | 0.1533 |  |
|  | 3.9072 | 0.1605 | 0.2398 | 0.1644 |  |
|  | 3.6958 | 0.1630 | 0.2300 | 0.1659 |  |
|  | 3.5155 | 0.1712 | 0.2028 | 0.1727 |  |
|  | 3.3591 | 0.1764 | 0.2325 | 0.1797 |  |
|  | 3.2220 | 0.1874 | 0.2229 | 0.1893 |  |
|  | 3.1003 | 0.1938 | 0.2167 | 0.1949 |  |
|  | 2.9915 | 0.1927 | 0.2419 | 0.1946 |  |
|  | 2.8934 | 0.2060 | 0.2498 | 0.2083 |  |
|  | 2.8044 | 0.2084 | 0.2345 | 0.2096 |  |
|  | 2.7231 | 0.2095 | 0.2717 | 0.2127 |  |
|  | 2.6484 | 0.2347 | 0.2998 | 0.2390 |  |
|  | 2.5796 | 0.2548 | 0.2796 | 0.2562 |  |
|  | **Overall** | 0.1884 | 0.2406 | 0.1910 |  |

# Rxpct factors in resolution bins at cycle 2


|  |  |  |  |  |  |  |  |  |
| --- | --- | --- | --- | --- | --- | --- | --- | --- |
|  | **<d> (Å)**  Rxpct\_Work | Rxpct\_Free | Rxpct\_All | |  |  |  |  |
|  | 16.1098 | 0.3920 | 0.4388 | 0.3936 |  |
|  | 9.3010 | 0.2332 | 0.2783 | 0.2359 |  |
|  | 7.2045 | 0.2024 | 0.2663 | 0.2059 |  |
|  | 6.0889 | 0.2042 | 0.2757 | 0.2074 |  |
|  | 5.3699 | 0.1614 | 0.2420 | 0.1647 |  |
|  | 4.8573 | 0.1493 | 0.1930 | 0.1518 |  |
|  | 4.4681 | 0.1509 | 0.2146 | 0.1541 |  |
|  | 4.1595 | 0.1492 | 0.2182 | 0.1532 |  |
|  | 3.9072 | 0.1605 | 0.2394 | 0.1643 |  |
|  | 3.6958 | 0.1631 | 0.2306 | 0.1660 |  |
|  | 3.5155 | 0.1711 | 0.2027 | 0.1726 |  |
|  | 3.3591 | 0.1763 | 0.2321 | 0.1796 |  |
|  | 3.2220 | 0.1872 | 0.2223 | 0.1892 |  |
|  | 3.1003 | 0.1935 | 0.2159 | 0.1946 |  |
|  | 2.9915 | 0.1928 | 0.2415 | 0.1946 |  |
|  | 2.8934 | 0.2061 | 0.2493 | 0.2084 |  |
|  | 2.8044 | 0.2084 | 0.2336 | 0.2095 |  |
|  | 2.7231 | 0.2099 | 0.2731 | 0.2131 |  |
|  | 2.6484 | 0.2352 | 0.3013 | 0.2395 |  |
|  | 2.5796 | 0.2557 | 0.2813 | 0.2572 |  |
|  | **Overall** | 0.1883 | 0.2404 | 0.1909 |  |

# Rxpct factors in resolution bins at cycle 3


|  |  |  |  |  |  |  |  |  |
| --- | --- | --- | --- | --- | --- | --- | --- | --- |
|  | **<d> (Å)**  Rxpct\_Work | Rxpct\_Free | Rxpct\_All | |  |  |  |  |
|  | 16.1098 | 0.3896 | 0.4353 | 0.3912 |  |
|  | 9.3010 | 0.2297 | 0.2812 | 0.2327 |  |
|  | 7.2045 | 0.1977 | 0.2741 | 0.2020 |  |
|  | 6.0889 | 0.1997 | 0.2728 | 0.2030 |  |
|  | 5.3699 | 0.1578 | 0.2479 | 0.1615 |  |
|  | 4.8573 | 0.1474 | 0.1931 | 0.1500 |  |
|  | 4.4681 | 0.1485 | 0.2177 | 0.1521 |  |
|  | 4.1595 | 0.1484 | 0.2140 | 0.1522 |  |
|  | 3.9072 | 0.1598 | 0.2384 | 0.1636 |  |
|  | 3.6958 | 0.1621 | 0.2297 | 0.1651 |  |
|  | 3.5155 | 0.1700 | 0.2029 | 0.1716 |  |
|  | 3.3591 | 0.1752 | 0.2356 | 0.1787 |  |
|  | 3.2220 | 0.1872 | 0.2245 | 0.1893 |  |
|  | 3.1003 | 0.1929 | 0.2149 | 0.1940 |  |
|  | 2.9915 | 0.1920 | 0.2465 | 0.1940 |  |
|  | 2.8934 | 0.2061 | 0.2460 | 0.2082 |  |
|  | 2.8044 | 0.2074 | 0.2304 | 0.2084 |  |
|  | 2.7231 | 0.2100 | 0.2736 | 0.2132 |  |
|  | 2.6484 | 0.2387 | 0.3023 | 0.2428 |  |
|  | 2.5796 | 0.2563 | 0.2862 | 0.2580 |  |
|  | **Overall** | 0.1866 | 0.2412 | 0.1894 |  |
